# The Cut-Off Values of Anthropometric Indices for Identifying Subjects at Risk for Metabolic Syndrome in Iranian Elderly Men

**DOI:** 10.1155/2014/907149

**Published:** 2014-03-23

**Authors:** Mojgan Gharipour, Masoumeh Sadeghi, Minoo Dianatkhah, Shirin Bidmeshgi, Alireza Ahmadi, Marzieh Tahri, Nizal Sarrafzadegan

**Affiliations:** ^1^Metabolic Syndrome Department, Isfahan Cardiovascular Research Center, Isfahan Cardiovascular Research Institute, Isfahan University of Medical Sciences, Isfahan, Iran; ^2^Cardiac Rehabilitation Research Center, Isfahan Cardiovascular Research Institute (WHO Collaborating Center), Isfahan University of Medical Sciences, Isfahan, Iran; ^3^Interventional Cardiology Research Center, Isfahan Cardiovascular Research Institute, Isfahan University of Medical Sciences, Isfahan, Iran; ^4^Hypertension Research Center, Isfahan Cardiovascular Research Institute, Isfahan University of Medical Sciences, Isfahan, Iran; ^5^Isfahan Cardiovascular Research Center, Isfahan Cardiovascular Research Institute, Isfahan University of Medical Sciences, Isfahan, Iran

## Abstract

*Aim*. This study aimed to investigate which anthropometric indices could be a better predictor of metabolic syndrome (MetS) and the cut-off points for these surrogates to appropriately differentiate MetS in the Iranian elderly. *Method*. The present cross-sectional study was conducted on a sample of Isfahan Healthy Heart Program (IHHP). MetS was defined according to Third Adult Treatment Panel (ATPIII). In total, 206 elderly subjects with MetS criteria were selected. Anthropometric indices were measured and plotted using receiver operating characteristic (ROC) curves. *Results*. WC followed by WHtR yielded the highest area under the curve (AUC) (0.683; 95% CI 0.606–0.761 and 0.680; 95% CI 0.602–0.758, resp.) for MetS. WC at a cut of 94.5 cm resulted in the highest Youden index with sensitivity 64% and 68% specificity to predict the presence of ≥2 metabolic risk factors. BMI had the lowest sensitivity and specificity for MetS and MetS components. WC has the best ability to detect MetS which followed by WHtR and BMI had a lower discriminating value comparatively. *Conclusion*. WC is the best predictor for predicting the presence of ≥2 metabolic risk factors among Iranian elderly population and the best value of WC is 94.5 cm. This cut-off values of WC should be advocated and used in Iranian men until larger cross-sectional studies show different results.

## 1. Introduction

Obesity increases the risk of cardiovascular disease in adults, has been strongly associated with insulin resistance in nondiabetic persons and in individuals with type 2 diabetes [1, 2], and is considered as prerequisite for the diagnosis of metabolic syndrome (MetS). Central obesity is defined using ethnicity-specific cut-off point of waist circumference (WC) [3]. In this regard, previous studies recommended the best cut-off points of WC values for prevention and control of cardiovascular disease in adults and children [4, 5]. Definition of obesity differs among various ethnics; therefore, many regional studies are conducted to find the best cut-off points for obesity [6]. The values of anthropometric indices are varied in different age groups with respect to race; for instance, the cut-off points of WC in studies in Oman, Iraq, and Korea were 80 cm, 97 cm, and 90 cm, respectively, in men and 84.5 cm, 99 cm, and 85 cm, respectively, in women [7]. Numerous national studies determined cut-off points for WC, WHR, and BMI among Iranian adolescents and adults [8–10]. For example, Iranian Multi-Centric Osteoporosis Studies (IMOS) [8] determined of WC in five major cities of Iran. Considering the dramatic changes in fat distribution and its function throughout life [11] and according to this fact these changes can have important consequences on the profile of risk factors for developing MetS [12]. Our previous study which was done to determine the prevalence of different phenotypes in various age groups demonstrated that current cut-off points for WC are not appropriate for distinguishing subjects at risk of developing MetS specially among Iranian elderly men [13]. We found that the prevalence of MetS decreased sharply in men above 65 years old, which is related to low prevalence of central obesity in this group. So this study is designed to determine appreciate cut-off points for anthropometric indices in the Iranian elderly especially in men.

## 2. Material and Methods

### 2.1. Study Population

Isfahan Healthy Heart Program is a comprehensive integrated community-based action-oriented study with a reference community which has been conducted by the Isfahan Cardiovascular Research Institute since 2000 and completed in 2007 [14, 15]. A random independent sample of adults was selected by multistage cluster sampling. The effect of confounding has been addressed by using random, stratified household sampling based on age and sex groups. The participants were more than 19 years old. The samples underwent a 30-minute interview by well-trained examiners to complete validated questionnaires containing questions on demography, socioeconomic status, smoking behavior, physical activity, nutritional habits, and other risk profiles. Informed consent was obtained from all subjects prior to their participation in this study, which was approved by the Ethical Committee of Isfahan University of Medical sciences. IHHP was covered under IRB protocol FW A00008578. In this substudy we consider only elder population (over 65) with MetS.

### 2.2. Data Collection

Information on sociodemographic factors and self-reported medical history were obtained by interview. Anthropometric measurements, including height, weight, and waist and hip circumferences, were taken with subjects wearing light clothing by well-trained examiners. Waist circumference was measured to the nearest 0.1 cm in the horizontal plane at the high point of the iliac crest during minimal respiration [15]. Blood pressure was measured with a mercury sphygmomanometer using right arms, in a sitting position, after a 5 min rest. Systolic and diastolic blood pressure were recorded twice and averages were used for the data analysis. Blood samples were drawn from an antecubital vein after an 8–12 hr overnight fast. Samples were stored at −20 until required for biochemical assays. Fasting venous blood samples were obtained from the antecubital vein between 08:00 and 09:30. Blood samples were centrifuged for 10 min at 906 g within 30 min of collection. Sera were analyzed for total cholesterol, high-density lipoprotein (HDL), triglycerides (TG), and fasting blood glucose (FBG). Low-density lipoprotein cholesterol (LDL) was calculated by the Friedewald equation when TG was less than 400 mg/dL [16]. TC was measured using enzymatic colorimetric methods. HDL was determined after dextran sulphate-magnesium chloride precipitation of HDL. All the tests were performed in the Central Laboratory of the Isfahan Cardiovascular Research Center and using autoanalyzer ELAN (Ependorf 2000). For quality control measures, this laboratory meets the criteria of the National Standard Laboratory (a WHO collaborating centre in Tehran).

### 2.3. MetS Definition

The ATPIII definition of MetS was met when three or more of the following criteria were present: waist circumference ≥ 102 cm; HDL < 40 mg/dL or specific treatment for this lipid abnormality; triglycerides ≥ 150 mg/dL or specific treatment for this lipid abnormality; systolic blood pressure ≥ 130 mmHg or diastolic blood pressure ≥ 85 mmHg or treatment of previously diagnosed hypertension; fasting glucose ≥ 100 mg/dL [14].

### 2.4. Statistical Analysis

Data entry was carried out using EPI Info. Data were analyzed using STATA software (Stata/IC 11.0, StataCorp LP, College Station, TX, USA). For all analyses, statistical significance was assessed at the level of 0.05 (2-tailed) and *P* value < 0.05 was considered as the borderline significance (marginal significance).

Receiver operating characteristic (ROC) analysis and the area under curve (AUC) were used to identify the sensitivity and specificity of anthropometric indices cut-off points for the detection of MetS without WC. The optimal cut-off values were defined as the point at which the value of “sensitivity + specificity − 1” was maximum (Youden's index). The Akaike information criterion (AIC) considered for comparing nonnested models and goodness of fit. Lower values of the index indicate the preferred model, that is, the one with the fewest parameters that still provides an adequate fit to the data.

## 3. Results

### 3.1. Baseline Characteristics

In total, 206 elderly subjects with metabolic syndrome were evaluated for this study. The mean age of participants was 71.85 ± 5.44. The mean of WC, BMI, WHR, and WHtR in the presence of MetS was 97.39 ± 10.63, 26.32 ± 3.96, 0.96 ± 0.05, 0.96 ± 0.05, and 58.55 ± 6.11. Subjects with MetS had both higher systolic and diastolic blood pressure than subjects without MetS (140.79 ± 19.70 and 81.84 ± 4.39 (*P* < 0.001) versus 124.12 ± 20.10 and 77.41 ± 10.78 (*P* < 0.023)) (Table 1).

### 3.2. Obesity Indices and Metabolic Syndrome Except MetS Using ROC Curves

**Figure 1:**
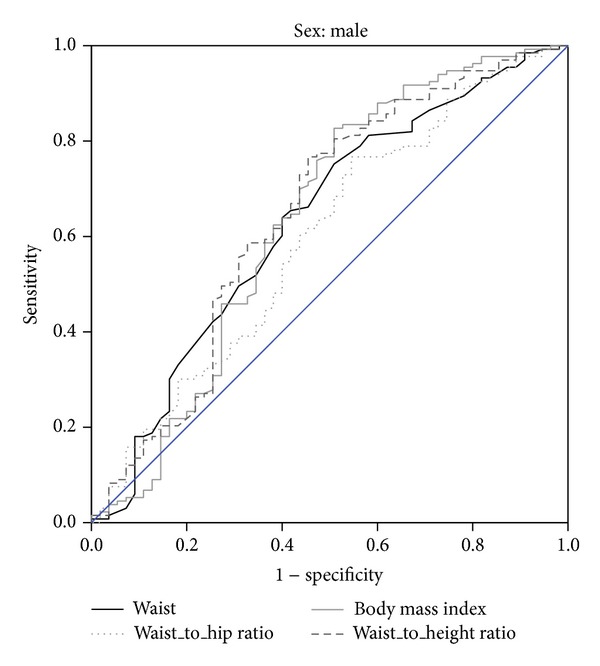
Receiver operating characteristics curve (ROC).

The predicting values for two or more metabolic risk factors and corresponding AUC of BMI, WC, WHR, and WHtR in men are shown in Table 2 and Figure 1. WC at a cut-off value of 94.5 cm resulted in the highest Youden index with corresponding sensitivity of 68% and 64% specificity to detect MetS. At a traditional cut-off value of <102 cm of WC, the sensitivity dropped to 88%, and specificity slightly rose to 23%.

The BMI at a cut-off value of ≥28 kg/m^2^ and the traditional cut-off value of ≥30 kg/m^2^ were found to be having the lowest Youden index and corresponding sensitivity and specificity.

These results showed that WC (with the AUC of 0.590) was better indicator for high blood pressure compared to BMI and WHR, whereas BMI (with the AUC of 0.638) was better indicator of high blood pressure and WHR (with the AUC of 0.595) had a lower discriminating value. WC had greater AUC values compared to BMI and WHR in distinguishing low HDL cholesterol and hypertriglyceridemia only (0.609 and 0.625, resp.). WHtR had better prediction ability to distinguish high blood pressure (AUC of 0.616 (0.527–0.705)).

Based on the indicator of interest, the optimum WC cut-off points in our study population for MetS were 94.5 and for MetS components ranged from 89.5 to 95.5 (Table 2). The best cut-off points of WHR for MetS were 0.95 and ranged from 0.92 to 0.97 for other MetS components. BMI at a cut-off value of the traditional cut-off value of 30 kg/m^2^ were found to be having the lowest Youden index and corresponding sensitivity and specificity.  Table 3 shows adjusted odds ratios (OR) for MetS. The model is adjusted for age and smoking status. The lowest AIC is related to WC in both adjusted and unadjusted model. So WC at a cut-off point of 94.5 cm happened to be the best predictor of MetS. In the presented cut-off of WC the risk of MetS increased by 3.84.

## 4. Discussion

The findings of the present study demonstrated the superlative discriminating values of common anthropometric parameters for MetS in elderly Iranian men. While many studies were done among adolescents and adults, but to the best of our knowledge this is the first study which was done on elder men. After considering Youden's index, we found that the best cut-off points for WC in men are 94.5 cm instead of 102 cm which is recommended by ATPIII.

In recent years numerous studies have been done to find the best anthropometric indices for detecting MetS, especially, among different ethnics. In our previous study [9] we showed that, among the Iranian population, WC might be the most appropriate indicator to discriminate MetS regardless of gender and age, which has been confirmed with the present study. In this study we found that the best obesity indicator for distinguishing MetS among Iranian elderly men is WC.

There is controversy about the proper values of anthropometric values according to ethnicity, genetic background, sexes, and sociocultural aspects. Beydoun et al. showed that WC is among the most powerful tools to predict MetS and that optimal cut-off points for various indices including WC may differ by sex and race [17]. Beydoun's suggestion is consistent with other reports by Reeder et al. [16, 18] and Wang et al. [19]. While many studies have been done to find the best similar to our results. For example, Wakabayashi and Daimon illustrated that the associations between obesity and multiple risk factors for atherosclerosis become weaker as age increases, while age does not influence cut-off values of obesity indices except for higher WHtR at an older age in women [18]. Another study which was done by Shao et al. indicated that WHtR might be an optimal anthropometric predictor of MetS risk factors and the cut-off point of WHtR was approximately 0.50 in both genders of Chinese adults [20]. Likewise, another report by Dong et al. suggested that WHtR has better association with obesity related cardiovascular risk conditions in both sexes, except for hypertension in Chinese men [21]. An Iranian longitudinal study confirmed the cut-off points for women but decreased them to 90 cm for men. This might be in part due to the linear effect of WC on cardiovascular risk in men compared to women. Hence, among all eleven cut-off points, 90 cm was identified as the best definition of central obesity for men [10]. An eastern study which was done on Japanese subjects reported that VFA was better than WC and BMI for identification of subjects with two or more components of MetS [22, 23]. Another Japanese study has shown that VFA does not have better correlation with carotid intima-media thickness as a surrogate measurement of atherosclerosis than waist-hip ratio or WC [24]. A study among Korean adult male population reported WC cut-off point of 86.5 cm which is obtained cut points [25]. These major differences in the cut-off values might be attributed to ethnic variations and using different criteria for diagnosing MetS. Seo et al. claimed that because WC is clinically practical measurement and is more cost beneficial than direct imaging which is required for assessing visceral fat, so they suggested that WC measurements are sufficient for the detection of central obesity in correlation with the risk of MetS in elderly Koreans [25].

In this study we found that WC at a cut-off value of 94.5 cm has the highest sensitivity and specificity to predict the presence of 2 or more metabolic risk factors. When we applied the WC cut-off value of ≥102 cm for men and ≥88 cm for women as recommended by ATPIII criteria [14], the sensitivity to discriminate between those with and without MetS dropped from 64% to 68%.

## 5. Limitation

The present study is limited by its cross-sectional nature, so we could not evaluate outcome measures. Consequently, the authors are mindful that differences could only be imputed from the previously documented data.

## 6. Conclusion

We found that BMI tended to be the weakest index for identifying MetS risk factors in elderly men. WC exhibited the best predictive index for MetS, almost similar to predictive powers of WHtR for identifying MetS. The two indices of WC and WHtR were better indicators of MetS. WC had WC had the highest sensitivity for MetS diagnosis among obesity indices. These cut-off values of WC should be advocated and used in Iranian elderly men.

## Figures and Tables

**Table 1:** Characteristics of study subjects according to the presence of two or more metabolic risk factors except for WC in the elderly Iranians.

	Men			
	Two or more metabolic risk factors of the NCEP-ATPIII of the criteria except for WC			
	Total	Absent	Present	*P* value
Age (years)	71.85 ± 5.44	72.15 ± 5.42	71.71 ± 5.49	0.568
Body mass index (kg/m2)	25.51 ± 4.02	24.36 ± 3.83	26.32 ± 3.96	0.001
WHR	0.94 ± 0.06	0.93 ± 0.06	0.96 ± 0.05	0.001
Waist (cm)	94.69 ± 11.10	90.86 ± 10.66	97.39 ± 10.63	0.000
WHtR	56.91 ± 6.51	54.56 ± 6.40	58.55 ± 6.11	0.000
Fasting blood sugar (mg/dL)	104.11 ± 37.67	90.12 ± 18.53	113.52 ± 44.14	0.000
Glucose (2hpp) (mg/dL)	124.45 ± 56.42	103.48 ± 28.21	140.74 ± 66.89	0.000
Triglycerides (mg/dL)	151.80 ± 87.78	101.25 ± 33.41	186.63 ± 96.41	0.000
HDL (mg/dL)	40.74 ± 10.64	48.08 ± 9.43	35.80 ± 8.31	0.000
Cholesterol (mg/dL)	199.56 ± 39.40	197.35 ± 39.38	200.68 ± 39.43	0.557
Systolic blood pressure	134.12 ± 21.40	124.12 ± 20.10	140.79 ± 19.70	0.000
Diastolic blood pressure	80.12 ± 13.22	77.41 ± 10.78	81.84 ± 4.39	0.023
Low HDL cholesterol (*n*, (%))	98 (48.5)	13 (15.9)	85 (70.8)	0.000
High TG (*n*, (%))	82 (40.8)	3 (3.7)	79 (66.4)	0.000
High blood pressure (*n*, (%))	136 (70.5)	35 (46.7)	101 (85.6)	0.000
High blood sugar (*n*, (%))	54 (26.6)	5 (6.1)	49 (40.5)	0.000
Lipid drug (*n*, (%))	24 (85.7)	5 (100.0)	19 (82.6)	1.000
Diabetes drug regular (*n*, (%))	30 (96.8)	5 (100)	25 (96.2)	1.000
Hypertension drug regular (*n*, (%))	38 (74.5)	5 (55.6)	33 (78.6)	0.150

The numerical values are presented as mean ± SD and compared by Student's *t*-test except for items indicated by § that Mann-Whitney *U *test was employed. Categorical data is shown as *n* (%) and tested by chi-square.

*BMI: body mass index, WC: waist circumference, WHR: waist-to-hip ratio, and WHtR: waist-to-height ratio.

^†^Triglycerides ≥ 150 mg/dL or on antilipid agents.

^††^HDL-C < 40 for men and <50 for women or on antilipid agents.

^‡^High blood pressure is considered as SBP ≥ 140 mmHg or DBP ≥ 90 mmHg or antihypertensive agents.

**Table 2:** Areas under the ROC curve of WC, BMI, WHR, and WHtR to identify the presence of the metabolic risk factors other than WC in elderly men.

	Obesity index	Best cut-off point	Sensitivity	Specificity	Youden	AUC (95% CI)	*P* value
MetS	WC	94.5	64%	68%	32%	0.683 (0.606–0.761)	0.000
	WHR	0.95	6%	69%	29%	0.645 (0.563–0.727)	0.001
	BMI	26.65	48%	76%	24%	0.641 (0.561–0.722)	0.001
	WHtR	58.66	52%	0.79	31%	0.680 (0.602–0.758)	

High blood pressure	WC	89.5	75%	49%	24%	0.633 (0.542–0.724)	0.004
	WHR	0.92	76%	45%	22%	0.595 (0.503–0.687)	0.040
	BMI	22.84	82%	49%		0.638 (0.540–0.735)	0.004
	WHtR	53.84	76%	54%	31%	0.616 (0.527–0.705)	

High triglyceride	WC	94.5	67%	59%	26%	0.625 (0.545–0.705)	0.003
	WHR	0.97	46%	72%	19%	0.585 (0.502–0.668)	0.020
	BMI	22.86	85%	35%	21%	0.600 (0.519–0.680)	0.04
	WHtR	53.09	0.85	0.38	0.22	0.607 (0.527–0.687)	

Low HDL	WC	94.5	63%	58%	21%	0.609 (0.527–0.690)	0.010
	WHR	0.96	52%	68%	20%	0.597 (0.516–0.679)	0.010
	BMI	24.83	63%	52%	15%	0.579 (0.498–0.661)	0.060
	WHtR	53.563	81%	40%	21%	0.604 (0.523–0.685)	

High fasting blood sugar	WC	95.5	61%	58%	19%	0.617 (0.524–0.709)	0.014
	WHR	0.95	65%	58%	24%	0.605 (0.518–0.691)	0.027
	BMI	25.54	58%	58%	18%	0.578 (0.482–0.673)	0.101
	WHtR	56.04	98%	15%	13%	0.512 (0.431–0.593)	

BMI: body mass index, WC: waist circumference, WHR: waist-to-hip ratio, and WHtR: waist-to-height ratio.

**Table 3:** Association of the best cut-offs of obesity indices with MetS.

	Crude OR (95% CI)			Adjusted OR (95% CI)		
	OR (CI)	*P* value	AIC	OR (CI)	*P* value	AIC
WC	3.835 (0.00–7.11)	0.000	**237.55**	4.564 (2.37–8.81)	0.000	240.19
WHR	3.00 (1.63–5.51)	0.000	243.81	3.219 (1.73–6.00)	0.000	248.26
BMI	2.954 (1.55–5.62)	0.001	247.1	3.115 (1.62–5.98)	0.001	251.78
WHtR	4.023 (2.07–7.80)	0.000	**238.121**	4.162 (2.13–8.12)	0.000	**243.09**

OR: odds ratio, CI: confidence interval, BMI: body mass index, WC: waist circumference, WHR: waist-to-hip ratio, and WHtR: waist-to-height ratio.

Model is adjusted for age and smoking status.
